# Advanced Stress Echocardiography with Cardiopulmonary Exercise Testing After Myocardial Infarction

**DOI:** 10.3390/jfmk10040393

**Published:** 2025-10-09

**Authors:** Nektarios Lampros Afthonidis, Vasiliki Michou, Maria Anyfanti, Anastasios Dalkiranis, George Panayiotou, Nikolaos Koutlianos, Evangelia Kouidi, Asterios Deligiannis

**Affiliations:** 1 Sports Medicine Laboratory, School of Physical Education and Sport Science, Aristotle University, 57001 Thessaloniki, Greece; nektafthonidis@gmail.com (N.L.A.); vasilikimichou@yahoo.gr (V.M.); manyfant@phed.auth.gr (M.A.); tdalkiranis@phed.auth.gr (A.D.); koutlian@phed.auth.gr (N.K.); kouidi@phed.auth.gr (E.K.); 2 Laboratory of Exercise, Health and Human Performance, Applied Sport Science Postgraduate Program, Department of Life Sciences, School of Sciences, European University Cyprus, 2404 Nicosia, Cyprus; g.panayiotou@euc.ac.cy

**Keywords:** global longitudinal strain, exercise capacity, left ventricular function, myocardial infarction, echocardiography, cardiopulmonary exercise testing

## Abstract

**Background:** A thorough post-myocardial infarction (MI) evaluation is essential for prognosis and rehabilitation. While cardiopulmonary exercise testing (CPET) is the standard for assessing functional capacity, combining it with dynamic stress echocardiography (DSE) may offer a more comprehensive assessment. **Aim:** This study examined the role of stress echocardiography (SE) in male post-MI patients by evaluating left ventricular function with conventional indices and the change in global longitudinal strain (ΔGLS) at rest and during maximal treadmill CPET. A secondary aim was to determine whether ΔGLS could provide additional value to traditional measures in post-MI care. **Methods:** Eighteen men with a recent MI [15 ST-elevation MI, three non-ST-elevation MI; mean age 53.2 ± 5.9 years, mean body mass index (BMI) 27.9 ± 2.2, 44.4% with a smoking history) and 18 age-matched male controls (mean age 50.1 ± 10.8 years, mean BMI 26.5 ± 2.4, 39.0% with smoking history) were enrolled. All MI patients were under optimal medical therapy, including β-blockers, which were withheld on the test day. Most underwent percutaneous coronary intervention (PCI), coronary artery bypass grafting (CABG) n = 2, or PCI for non-ST-elevation MI (NSTEMI) n = 3. Left ventricular ejection fraction (LVEF) and global longitudinal strain (GLS) were measured at rest and at peak effort and correlated with CPET parameters. **Results:** Post-MI patients had lower LVEF (50.6% vs. 60.7% at rest; 55.3% vs. 67.4% at peak, both *p* < 0.001), impaired GLS (–14.7% vs. –20.2% at rest, *p* = 0.003; –15.8% vs. –22.7% at peak, *p* = 0.001), and reduced VO_2_peak (29.2 vs. 41.9 mL/kg/min, *p* < 0.001) compared with controls. In the MI group, ΔGLS correlated with VO_2_peak (r = –0.645, *p* = 0.003) and VE/VCO_2_ (r = 0.539, *p* = 0.020), indicating its potential as a marker of functional reserve. **Conclusions:** Combined CPET and SE offered comprehensive insights into functional and myocardial performance, identifying ΔGLS as a useful non-invasive index for risk stratification and rehabilitation after MI, with high feasibility and safety.

## 1. Introduction

Ischemic heart disease, often following myocardial infarction (MI), remains the leading cause of mortality worldwide. Although its incidence has declined in Europe, it still accounts for about 20% of all deaths [1]. Reported international incidence rates of MI range between 43 and 144 cases per 100,000 individuals annually [2,3]. In Greece, approximately 20,000 new cases are diagnosed each year. The distribution between ST-elevation MI (STEMI) and non-ST-elevation MI (NSTEMI) has shifted in recent decades, with NSTEMI becoming increasingly frequent [4,5]. MI results in structural and functional alterations of the heart, impaired autonomic control, reduced exercise capacity, and elevated mortality risk [6,7]. It also creates a considerable socioeconomic burden, mainly through ischemic heart failure, which remains a leading source of morbidity and mortality [3,8]. Consequently, accurate post-MI evaluation is essential, including defining infarct type and extent, quantifying global and regional function, and assessing exercise capacity. Advances in non-invasive imaging have enhanced the precision of these assessments [9,10,11].

Sex differences play an important role in the epidemiology and clinical presentation of myocardial infarction. Women represent about 30–40% of acute MI cases and are 20–30% less likely than men to present with STEMI, being more frequently diagnosed with NSTEMI [12,13,14]. The incidence of STEMI is up to five times higher in men than in women aged 35–54 years, whereas this difference narrows considerably in older populations [13]. Additionally, in patients with STEMI, sex differences also extend to clinical outcomes and time-sensitive care. An Italian cohort study found that female sex, older age, prior IM, and night-time interventions were independently associated with higher in-hospital mortality [15]. Beyond acute care, differences have also been examined in post-MI cardiac remodeling. In the PARADISE-MI echocardiographic sub study, women exhibited higher baseline left ventricular ejection fraction (LVEF), and smaller ventricular volumes compared with men, but longitudinal changes in chamber size and function, as well as the prognostic association between echocardiographic parameters and adverse outcomes, did not differ significantly by sex [16]. Genetic and molecular mechanisms are also increasingly recognized as important contributors to myocardial infarction and subsequent remodeling. Xu et al. [17] showed that methylprotodioscin improved cardiac function and reduced infarct size via COX6C regulation. Similarly, Wang et al. [18] identified thymic stromal lymphopoietin (TSLP) as a key regulator of cardiac repair after MI. While Sumi et al. [19] reviewed how DNA methylation and histone modifications (such as methylation, demethylation and acetylation), together with non-coding RNAs, contribute to disease onset and progression, and importantly showed how environmental exposures (such as smoking, alcohol use, air pollution, and dietary factors) and lifestyle behaviors (including physical inactivity, obesity, and metabolic stress) can modulate these epigenetic processes and thereby influence risk. These studies highlight molecular pathways that influence outcomes after MI and suggest potential therapeutic targets. 

The current gold standard for clinical monitoring before and after MI follows guideline-directed pathways that integrate resting and stress echocardiography (SE), cardiopulmonary exercise testing (CPET) where indicated, biomarkers, and when available, cardiac magnetic resonance (CMR) for infarct characterization and viability, alongside risk-factor optimization and cardiac rehabilitation [1,4,20].

The integration of dynamic echocardiography (DE) with CPET has gained attention. Stress echocardiography combined with exercise testing has been applied in heart failure [21,22,23,24,25,26], hypertension [24,27], and Coronary Artery Disease (CAD) [28,29], but data specifically in post-MI patients are limited [30]. LVEF remains the traditional cornerstone for cardiac evaluation in heart failure [20], yet it correlates poorly with exercise capacity and suffers from interobserver variability [20,30,31,32]. Advanced indices, particularly global longitudinal strain (GLS), may provide greater sensitivity and prognostic relevance. Prior work has linked resting GLS to exercise capacity in CAD [33] and assessed exercise limitation in acute MI survivors using semi-supine ergometry [30]. However, semi-supine protocols can limit maximal workload relative to treadmill testing.

Therefore, we aimed to evaluate left ventricular morphology and function using DE (at rest and immediately post-exercise) in combination with maximal treadmill CPET in male post-MI patients, and to investigate correlations between imaging parameters (including ΔGLS) and functional performance. We hypothesized that ΔGLS would be significantly associated with exercise performance, complementing traditional indices, and providing a practical non-invasive marker for post-MI assessment and rehabilitation planning.

## 2. Materials and Methods

### 2.1. Study Design

This prospective case–control study was performed at the Sports Medicine Laboratory, Aristotle University of Thessaloniki. All participants underwent a structured protocol that included a medical history, anthropometric assessment, blood pressure measurement, a resting 12-lead electrocardiogram, maximal treadmill CPET, and DE performed at rest and at peak exercise. Ethical approval was obtained from the Ethics Committee of the Aristotle University of Thessaloniki (Protocol 49/2021), and all participants provided written informed consent. The study adhered to the Declaration of Helsinki. Recruitment for the trial started in January 2022 and ended in November 2023.

### 2.2. Participants

Participants were recruited through an open invitation in the Thessaloniki prefecture, Greece. Eligibility criteria for the MI group included: documented MI within the preceding six months (≥4 weeks prior to enrollment), classified as either ST-elevation MI (STEMI) or non-ST-elevation MI (NSTEMI) based on ECG and biomarker findings, male sex, age >18 years, well-controlled blood pressure for at least two weeks, absence of diabetes mellitus, orthopedic or neurological limitations, and absence of thyroid dysfunction. An adequate echocardiographic acoustic window was also required. Exclusion criteria were female sex, age <18 years, unstable angina, malignant arrhythmias, uncontrolled heart failure, significant valvular disease, severe left ventricular dysfunction (LVEF < 30%), or inadequate image quality (defined as >1 non-visualized segment). Sociodemographic and lifestyle information (e.g., smoking history) was collected via standardized questionnaire. Anthropometric/body-composition variables [height, weight, body mass index (BMI)] were measured in all participants.

All patients received guideline-directed medical therapy, including β-blockers, angiotensin-converting enzyme inhibitors (ACEIs) or angiotensin receptor blockers (ARBs), statins, and antiplatelet agents. To avoid acute pharmacological interference with exercise testing, β-blockers were withheld on the day of CPET and DE but resumed immediately afterwards. The control group met identical inclusion and exclusion criteria, except for the absence of an MI history. Among the 18 MI patients, 15 (83.3%) had STEMI and 3 (16.7%) had NSTEMI. Two underwent coronary artery bypass grafting (CABG), while the remainder were treated with percutaneous coronary intervention (PCI): left anterior descending artery (n = 8), right coronary artery (n = 5), NSTEMI PCI (n = 3).

### 2.3. Sample Size Calculation

To determine the minimum required sample, we performed a priori sample-size calculation (IBM SPSS v27, IBM, Armonk, NY, USA) for a two-tailed test with an alpha level (α) of 0.05 and power = 80%. The required total sample was 34 participants (17 per group). For n = 34, the calculated power was 0.802 with a Type I error of 5% and a Type II error (β) of 0.198. Ultimately, we enrolled 36 participants, with 18 assigned to each group.

### 2.4. Cardiopulmonary Exercise Testing

Symptom-limited CPET was conducted on a treadmill (T2100-ST1 Treadmill, GE HealthCare, Milwaukee, WI, USA) Technologies Inc using the standard Bruce protocol [34], validated for both CAD and post-MI populations [35]. All tests were scheduled between 08:00 and 11:00 a.m. to minimize circadian variation. A brief warm-up (light walking) preceded each test. The Bruce protocol consisted of 3-min stages with progressive increases in both treadmill speed and incline, inducing a stepwise increase in external workload until maximal effort or symptom limitation. Continuous ECG monitoring (GE Medical Systems, Milwaukee, WI, USA) and stage-wise blood pressure assessment were performed. Gas exchange variables were measured breath-by-breath using a metabolic cart (MedGraphics Breeze Suite CPX Ultima, Milwaukee, WI, USA) and PREVENT® face mask (St. Paul, MN, USA). The system was calibrated before each test per manufacturer instructions. 

Analyzed parameters included peak oxygen uptake (VO_2_peak), ventilation (VE), and ventilatory efficiency (VE/VCO_2_ slope). Exercise tests were terminated when one or more of the following were achieved: (1) respiratory exchange ratio ≥1.10, (2) plateau in oxygen uptake despite increasing workload, (3) volitional exhaustion with a rating of perceived exertion ≥18 on the Borg scale, or (4) achievement of ≥85% of age-predicted maximal heart rate (220–age) [36,37]. For all patients, β-blockers were withheld on the test day and resumed immediately afterwards.

### 2.5. Dynamic Echocardiography

DE was performed at rest and immediately after peak exercise (within <60 s of treadmill cessation) using a Vivid S70 system (GE Medical, Horten, Norway). Images were obtained in apical four-, two-, and three-chamber views, as well as parasternal short-axis views. Although semi-supine ergometry can facilitate imaging, treadmill testing was selected to ensure maximal workload and accurate VO_2_peak assessment. Two experienced cardiologists, blinded to clinical data, performed offline analysis using EchoPAC (v204, GE Healthcare). Additionally, the statistical team was blinded to group allocation to minimize analytic bias. LVEF was calculated using the modified Simpson biplane method [38]. GLS was derived from 2D speckle-tracking. ΔLVEF and ΔGLS were calculated as peak minus rest values. Analyses followed international guidelines [38,39].

### 2.6. Statistical Analysis

Data analysis was performed using IBM SPSS v27 (IBM, Armonk, NY, USA). The Shapiro–Wilk test was used to assess normality. As all variables were normally distributed, continuous data are presented as mean ± SD, and parametric tests were applied. Between-group comparisons used independent-samples Student’s *t*-tests. Effect sizes were calculated for all comparisons using Cohen’s d for *t*-tests (with thresholds of 0.2, 0.5, and 0.8 indicating small, medium, and large effects, respectively) and Cramer’s V for categorical variables. Intra-observer reproducibility was assessed using intraclass correlation coefficients (ICC) calculated with a two-way mixed-effects model, absolute agreement, average measures [ICC(3,k)]. Pearson correlation examined associations between CPET variables (VO_2_peak, VE/VCO_2_) and echocardiographic indices (LVEF, GLS, ΔGLS). Simple linear regression was performed to model these associations, and the regression equations (y = β_0_ + β_1_x), together with r and *p* values, are presented in the figures. A post hoc power calculation for the primary outcome (between-group ΔGLS) indicated power >80% at α = 0.05. Statistical significance was set at *p* < 0.05.

## 3. Results

### 3.1. Participants’ Characteristics

**Figure 1:**
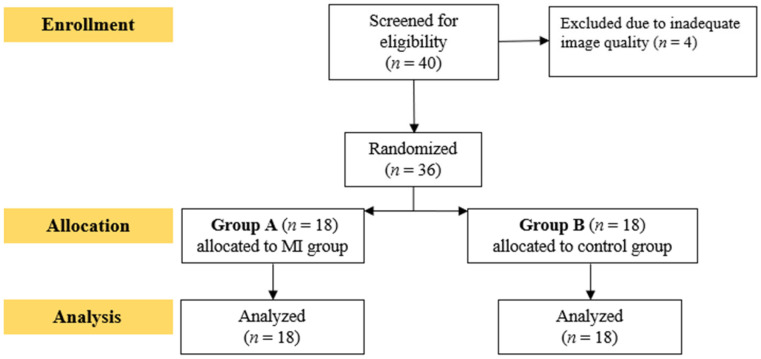
Flowchart of participants recruitment.

Of the 40 individuals initially enrolled, four (two MI patients and two controls) were excluded due to inadequate image quality, leaving a final sample of 36 participants (Figure 1). Among the MI group (n = 18), 15 (83.3%) had STEMI and 3 (16.7%) had NSTEMI. Two patients underwent CABG, while 13 were treated with PCI (left anterior descending artery, n = 8; right coronary artery, n = 5; NSTEMI PCI, n = 3). All patients were receiving optimal medical therapy, including β-blockers, ACEIs/ARBs, statins, and dual antiplatelet therapy, with β-blockers withheld on the test day. Baseline characteristics are summarized in Table 1. Groups did not differ significantly in age, BMI, blood pressure, or smoking history. Resting LVEF was significantly lower in the MI group.

### 3.2. Cardiorespiratory Efficiency Results

Compared with controls, MI patients had reduced exercise duration (–18.5%, *p* = 0.002), lower peak exercise heart rate (–8.3%, *p* = 0.036) and markedly reduced VO_2_peak (29.2 ± 6.1 vs. 41.9 ± 7.4 mL/kg/min, *p* < 0.001). VE/VCO_2_ slope was higher in the MI group (*p* = 0.018), indicating impaired ventilatory response (Table 2). 

### 3.3. Echocardiographic Findings

**Figure 2:**
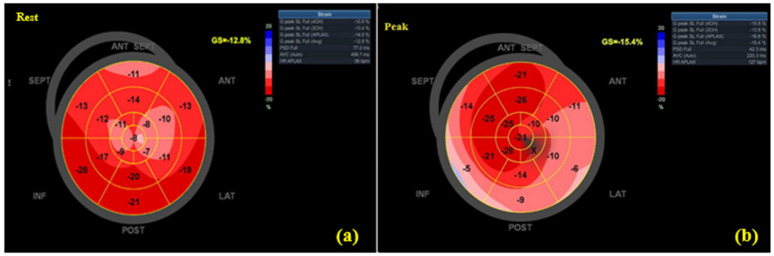
GLS bull’s-eye plots at rest (**a**) and peak (**b**) in a recent-MI patient (absolute GLS decreases at peak).

**Figure 3:**
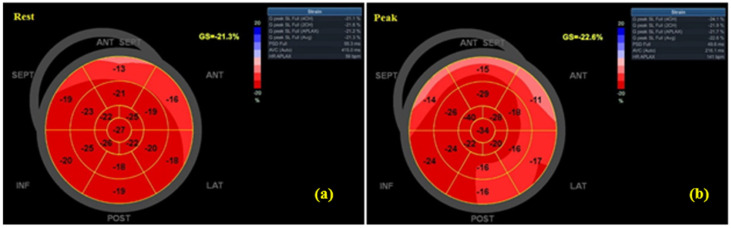
GLS bull’s-eye plots at rest (**a**) and peak (**b**) in a healthy control (absolute GLS increases at peak).

At rest, the MI group demonstrated lower LVEF (50.6% ± 4.8 vs. 60.7% ± 5.6, *p* < 0.001) and less negative GLS (–14.7% ± 2.9 vs. –20.2% ± 3.2, *p* = 0.003). At peak exercise, differences widened: LVEF (55.3% ± 5.1 vs. 67.4% ± 6.3, *p* < 0.001) and GLS (–15.8% ± 3.1 vs. –22.7% ± 2.8, *p* = 0.001) (Table 3). The magnitude of functional reserve (ΔLVEF, ΔGLS) was smaller in MI patients, reflecting impaired adaptability. Feasibility was high, with adequate segment visualization in 97.7% of participants. Intra-observer reproducibility was excellent (ICC(3,k) = 0.94, 95% CI 0.88–0.97) for GLS. Figure 2 and Figure 3 show GLS bull’s-eye plots at rest and during exercise from a recent-MI patient and a healthy control, respectively. 

### 3.4. Correlations Between CPET and Echocardiographic Parameters

**Figure 4:**
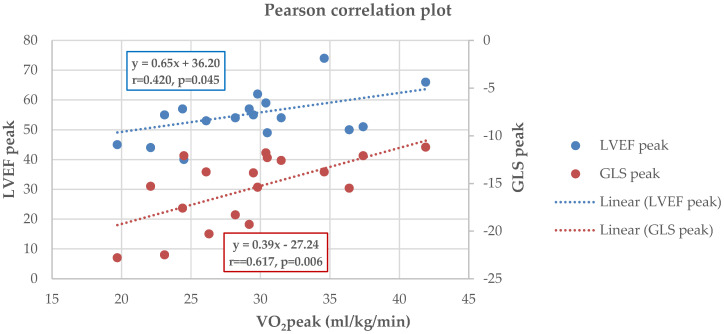
Pearson analysis between VO_2__peak_ and GLS_peak_ (r = 0.617, *p* = 0.006) and VO_2__peak_ and LVEF_peak_ (r = 0.420, *p* = 0.045) in the MI group; regression lines displayed with equations y = β_0_ + β_1_x.

**Figure 5:**
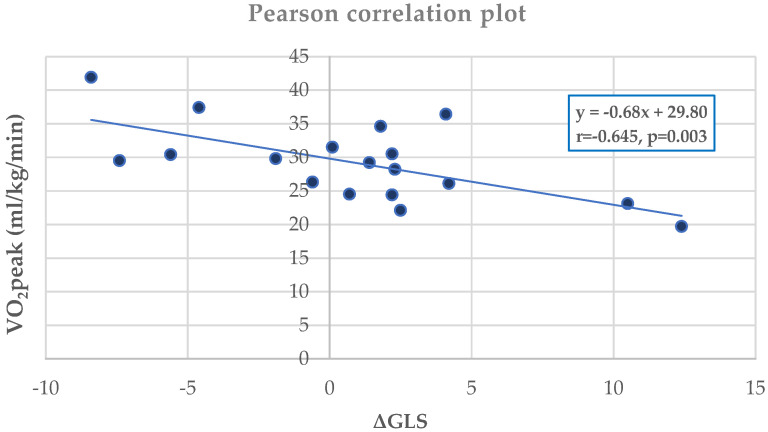
Pearson analysis between VO_2_peak and ΔGLS (r = –0.645, *p* = 0.003) in the MI group, with regression line and equation.

**Figure 6:**
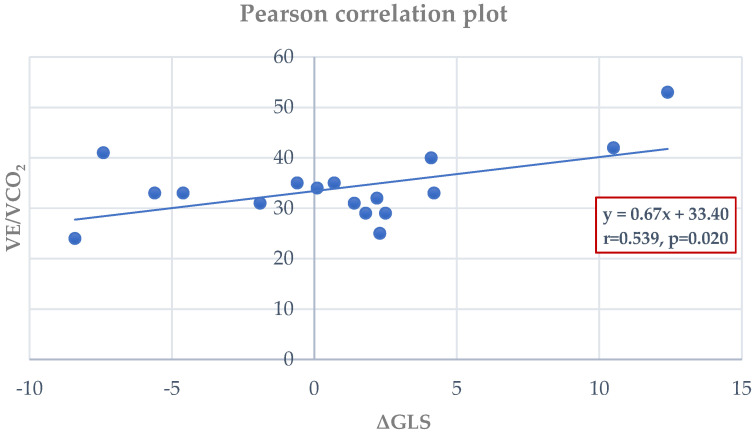
Pearson analysis between VE/VCO_2_ slope and ΔGLS (r = 0.539, *p* = 0.020) with regression line and equation.

Within the MI group, VO_2_peak correlated significantly with peak LVEF (r = 0.420, *p* = 0.045) (Figure 4) and peak GLS (r = 0.617, *p* = 0.006) (Figure 4). ΔGLS showed a strong negative correlation with VO_2_peak (r = –0.645, *p* = 0.003) (Figure 5) and a positive correlation with VE/VCO_2_ slope (r = 0.539, *p* = 0.020) (Figure 6). No such correlations were observed in controls.

## 4. Discussion

This study evaluated the combined application of maximal treadmill CPET and DE in men with recent MI. The approach was feasible, safe, and informative. Simultaneous assessment of cardiac mechanics and exercise capacity under physiological stress yields insights beyond resting measurements and helps detect subtle myocardial dysfunction. Our findings align with prior work demonstrated persistent systolic impairment in MI survivors despite preserved exercise reserve [30,40,41]. The smaller ΔGLS observed in MI patients indicates reduced contractile adaptability, aligning with previous reports that GLS is sensitive to afterload and subclinical dysfunction [42,43,44]. Caunite et al. [45] demonstrated that in STEMI patients, a relative reduction in GLS exceeding 7% at one-year follow-up was independently associated with an approximately twofold increase in the risk of all-cause mortality, whereas patients who exhibited GLS improvement or only a nonsignificant decline experienced substantially better long-term survival. Controls exhibited more negative GLS at peak (greater absolute magnitude), reflecting preserved adaptability. ΔGLS correlated strongly with VO_2_peak and ventilatory efficiency (VE/VCO_2_), suggesting its clinical utility for risk stratification and exercise prescription. Unlike LVEF, which has variability and limited sensitivity, ΔGLS offers a reproducible index that integrates mechanical performance with functional capacity.

Our combined treadmill-CPET and exercise SE protocol was feasible and safe. Both LVEF and GLS increased at peak in both groups yet remained significantly worse in MI patients. Minimal ΔGLS in healthy controls is plausible given preserved myocardial function and afterload-dependence of GLS [43,46]. Sex-based differences in LVEF/GLS have been reported (women often show higher LVEF and more negative GLS at comparable workloads), likely due in part to lower SBP/afterload [47,48,49], though Larsen et al. [50] found no sex differences during bicycle testing. These observations underscore the importance of considering sex-specific physiology in clinical assessments.

Compared with Smarz et al. [30], using semi-supine ergometry, our tread-mill-based protocol prioritized physiological workload and accurate VO_2_peak. Although imaging occurred within <60 s post-peak, which may slightly underestimate peak systolic function, feasibility was high (97.7% visualization) and reproducibility excellent. No adverse events occurred [e.g., Ventricular Tachycardia (VT)/Ventricular Fibrillation (VF), syncope, high-grade atrioventricular (AV) block, or recurrent MI), supporting safety and aligning with international data [51,52]. The chosen protocol allowed imaging at rest, immediately post-exercise (peak), and recovery; on-treadmill imaging was not attempted.

The treadmill CPET test, with its long history as a diagnostic tool in subjects with suspected CAD and patients with known CAD [53], plays a crucial role in guiding their management and providing prognostic information. While CPET is not a routine test in these patients, it has the potential to significantly enhance our understanding of their condition by providing additional diagnostic and prognostic information [54,55]. In a study by Mazaheri et al. [56], 31 men with CAD-suspicious symptoms underwent a CPET followed by coronary angiography. The results indicated that a significant VE/VCO2 ratio change can predict exercise-induced MI. In addition, CPET is useful in assessing patients with ischemic heart disease, since the VO2/HR ratio often shows an early plateau or even a drop in value, indicating a pathological response of the pulse volume during the test [57].

Regarding the significance of CPET in MI evaluation, CPET plays a pivotal role in guiding the initial assessment of patients following an MI and in planning an effective and safer rehabilitation program. This reassures the medical community about the program’s safety and effectiveness, promoting its use [58]. Our study revealed that patients with recent MI had significantly lower VO_2_peak scores, and a non-significantly higher VE/VCO2 ratio, than healthy controls. In a groundbreaking observational study by Satoh et al. [59], it was noticed that a significant number of patients who experienced acute MI exhibited excessive ventilation, which improved within four months. The improvement was more pronounced in patients with higher levels of excessive ventilation. However, this improvement did not correspond to an enhancement in exercise capacity or hemodynamics.

Furthermore, DE is a valuable tool in patients with a history of MI and ischemic heart disease. It primarily quantifies the left ventricular systolic reserve, providing a predictor of adverse outcomes [60]. The absence of systolic reserve is linked to an in-creased risk of future events, cardiac death, and death from any cause [60]. During exercise, DE imaging can be challenging. The intense respiratory movements that occur during image acquisition immediately after maximum exertion complicate the process, resulting in a small percentage of the 18 myocardial segments needing to be adequately visualized (a 97.71% success rate in strain recording at peak exertion). However, over-all, the studies had sufficient image quality to calculate LVEF and GLS values at rest and during maximum exercise. These rates were higher than those reported by Caniggia et al. [61]. In that study, suboptimal acoustic windows were not listed as exclusion criteria for participants, and both the exercise and DE protocols differed from those used in the current study. In contrast, our rates were more aligned with those reported by Leitman et al. [62], who excluded suboptimal patients a priori. They followed a protocol similar to ours, which involved exercise on a treadmill and ultrasound imaging performed in the left lateral position during the first minute after maximum exercise. Our findings support the incorporation of GLS and ΔGLS into post-MI evaluations to complement LVEF, providing a more comprehensive assessment of functional reserve.

Specifically, in individuals following a ΜΙ, there is a shortage of data on the relationship between ultrasound parameters and patients’ functional status. Our study revealed a positive association between GLS at peak exercise and VO2peak, LVEF at peak exercise and VO2peak and between VE/VCO2 and ΔGLS. Conversely, we observed a negative correlation between ΔGLS and VO2peak. These findings are poised to contribute to more in-depth exploration and optimal management of such patients. The integration of ΔGLS into clinical assessment could enhance the precision of post-MI evaluation in several ways: (1) guide exercise intensity by identifying limited contractile reserve; (2) enhance prognostic stratification by linking myocardial deformation with ventilatory efficiency; and (3) complement LVEF, particularly when LVEF is preserved or mid-range yet functional impairment exists.

Beyond echocardiographic, functional measures, age and sex, other clinical and biological factors are also important to consider in the evaluation of post-MI patients. For instance, data from the UK Biobank demonstrated that elevated resting heart rate (RHR) is an independent predictor of future STEMI, with values >90 bpm conferring nearly a threefold increased risk, underscoring the importance of autonomic regulation as a prognostic marker [63]. Furthermore, evidence from the JACR Registry highlights the role of comprehensive secondary prevention, showing that cardiac rehabilitation not only improves exercise capacity but also favorably modifies cardiovascular risk factors in ACS patients [64]. In addition, early-onset ASCVD studies emphasize the contribution of traditional and sex-specific risk factors, including smoking, hypertension, lipid abnormalities, diabetes, and pregnancy-related complications, as well as emerging indices such as apolipoprotein B, lipoprotein(a), and coronary calcium in refining long-term risk prediction [65]. In a recent study by Zhang et al. [66], showed that patients diagnosed with coronary slow flow (CSF) had higher BMI, smoking prevalence, hyperuricemia, peripheral arterial disease, lower high-density lipoprotein cholesterol (HDL-C), and elevated triglycerides, compared to healthy controls. These patients had also demonstrated significantly impaired GLS, highlighting subclinical systolic dysfunction detectable by CMR.

Epigenome-wide association studies have identified DNA methylation loci linked to AMI, particularly in pathways of smoking, lipid metabolism, and inflammation, although their predictive capacity beyond conventional risk scores remains limited [67]. In STEMI patients with acute heart failure, (neutrophil extracellular traps) NETs markers such as dsDNA (double-stranded DNA) and MPO-DNA (myeloperoxidase–DNA complexes) showed modest correlations with interleukin-8 levels, but not with GLS or LVEF. Mixed-model regression did not confirm a causal role, suggesting NETs may reflect inflammatory stress rather than directly driving myocardial recovery [68].

To summarize, the current study acknowledges both the strengths and limitations. Strengths of the study include its prospective design, standardized imaging and CPET protocols, and blinded analysis by both cardiologists and statisticians. The addition of a post-hoc power calculation confirmed that the study was adequately powered for the primary endpoint (ΔGLS). Our study has also revealed meaningful correlations between CPET and DSE parameters in post-MI patients, shedding light on the potential diagnostic and prognostic value of combining these methods in the evaluation and management of MI. This finding has the potential to significantly impact patient care and assessment. Furthermore, the successful use of advanced DSE techniques, such as GLS and ΔGLS, for a more comprehensive evaluation of patients with cardiovascular disease is a promising development that can inspire future studies in this field.

On the contrary, this research has also certain limitations. Firstly, it was conduct-ed at a single center and encompassed participants from a specific city in Greece. Secondly, only male participants were included (chosen to optimize recovery-phase im-aging), as echocardiographic imaging during recovery is technically more challenging in women. While this choice enhanced data quality, it reduces generalizability, and future studies must include female patient, as SE has demonstrated consistent diagnostic and prognostic accuracy across both sexes [69]. Thirdly, sample size was modest (18 MI patients and 18 controls), restricting statistical power for subgroup analyses. This limitation reflects the pilot nature of the study, which was designed primarily to establish feasibility. Fourthly, the short delay in post-exercise image acquisition may have underestimated peak values, although this was minimized by rapid imaging (<60 s). Fifth, the study design was cross-sectional, with no longitudinal follow-up to assess prognostic outcomes. Sixth, neither the MI patients nor the controls underwent biochemical testing, which would have allowed a more detailed characterization and comparison of health status between groups. Finally, although baseline factors such as BMI, smoking, and blood pressure did not differ significantly between groups, these variables may still have contributed as potential confounders. Larger, multicenter studies with multivariable adjustment are needed.

## 5. Conclusions

The present study demonstrates that the combined use of treadmill CPET and DE is a feasible and safe approach for evaluating male patients after MI. Both LVEF and GLS were significantly impaired in the MI group at rest and peak exercise, and ΔGLS was closely associated with VO_2_peak and VE/VCO_2_. These findings indicate that ΔGLS provides added value beyond conventional parameters in the functional assessment of post-MI patients and may support exercise-based rehabilitation strategies.

## Figures and Tables

**Table 1:** Clinical characteristics of participants.

	MI-Group (n_A_ = 18)	Healthy Controls (n_B_ = 18)	Effect Size (Cohen’s d/Cramer’s V)	*p*-Value
Age (years)	53.22 ± 5.93	50.11 ± 10.77	0.36 ^d^	*p* = 0.291
BMI (kg/cm^2^)	27.96 ± 2.24	26.59 ± 2.42	0.59 ^d^	*p* = 0.090
STEMI				
Yes	10 (55.55%)	-	-	-
No	8 (44.44%)	-	-	-
NSTEMI				
Yes	8 (44.44%)	-	-	-
No	10 (55.55%)	-	-	-
PCI				
Yes	8 (44.44%)			
No	10 (55.55%)			
CABG				
Yes	3 (16.66%)			
No	7 (38.88%)			
Smoking history				
Yes	8 (44.44%)	7 (39.00%)	OR = 1.26 (95% CI: 0.33–4.74), Cramer’s V = 0.06	*p* = 0.744
No	10 (55.56%)	11 (61.00%)		*p* = 0.823
HRrest (bpm)	75.16 ± 12.34	76.27 ± 11.88	0.09 ^d^	*p* = 0.874
SBPrest (mmHg)	119.72 ± 9.62	125.55 ± 9.68	0.58 ^d^	*p* = 0.078
DBPrest (mmHg)	72.77 ± 4.91	75.55 ± 11.61	0.31 ^d^	*p* = 0.651

Note: Cohen’s d (^d^) was used for continuous variables. Odds ratio (OR) and Cramer’s V for categorical variables. CI: Confidence Interval (lower bound/upper bound); ΒΜΙ; body mass index; STEMI: ST elevation myocardial infraction; PCI: percutaneous coronary intervention; CABG: coronary artery bypass graft; HR: heart rate; SBP: systolic blood pressure; DBP: diastolic blood pressure; Data are mean ± SD; *p* < 0.05: ΜΙ group vs. healthy controls.

**Table 2:** Cardiorespiratory efficiency results.

	MI Group (n_A_ = 18)	Healthy Controls (n_B_ = 18)	Effect Size (Cohen’s d)	*p*-Value
Exercise time (min)	7.80 ± 1.67	9.57 ± 1.87	0.79	*p* = 0.002
VO_2_peak (mL/kg/min)	29.20 ± 5.74	41.92 ± 13.22	0.98	*p* = 0.0006
VE/VCO_2_	34.11 ± 4.10	30.33 ± 6.83	0.67	*p* = 0.114
RERmax	1.15 ± 0.16	1.22 ± 0.22	0.36	*p* = 0.295
HRmax (bpm)	149.33 ± 18.99	162.77 ± 17.99	0.73	*p* = 0.036
SBPmax (mmHg)	155.27 ± 15.76	165.27 ± 17.69	0.60	*p* = 0.082
DBPmax (mmHg)	72.77 ± 6.90	75.00 ± 10.71	0.25	*p* = 0.772

Note: VO_2_peak: maximum oxygen consumption; VE/VCO_2_max: ventilatory equivalents for carbon dioxide; RER: Respiratory exchange ratio; HR: Heart rate; SBP: Systolic blood pressure; DBP: Diastolic blood pressure; Data are expressed as mean ± SD; *p* < 0.05: ΜΙ group A vs. healthy controls.

**Table 3:** Dynamic echocardiography results.

	MI Group (n_A_ = 18)	Healthy Controls (n_B_ = 18)	Effect Size (Cohen’s d)	*p*-Value
LVEF rest (%)	50.61 ± 6.51	60.72 ± 4.33	0.85	*p* < 0.001
LVEF peak (%)	55.33 ± 8.87	67.44 ± 4.17	0.88	*p* < 0.001
GLS rest (%)	−14.70 ± 3.81	−20.22 ± 1.45	0.72	*p* = 0.003
GLS peak (%)	−15.78 ± 3.61	−22.72 ± 1.71	0.76	*p* = 0.001

Note: LVEF: Left Ventricular Ejection Fraction; GLS: Global Longitudinal Strain; data are expressed as mean ± SD; *p* < 0.05: ΜΙ group A vs. healthy controls.

## Data Availability

The data presented in this study is available on request from the corresponding author. The data is not publicly available due to ethical restrictions.
